# Not a Statin-Induced Myopathy: Metastatic Pancreatic Adenocarcinoma Presenting As Paraneoplastic Myositis

**DOI:** 10.7759/cureus.25016

**Published:** 2022-05-15

**Authors:** Gonca Ozcan, Anjiya Shaikh, Erica Becker, Nikola Perosevic

**Affiliations:** 1 Internal Medicine, University of Connecticut Health, Farmington, USA; 2 Internal Medicine, Saint Francis Hospital, Hartford, USA

**Keywords:** cancer-associated myositis, polymyositis, myopathy, pancreatic cancer, paraneoplastic myositis, metastatic pancreatic adenocarcinoma

## Abstract

Polymyositis is an inflammatory disease that causes bilateral proximal muscle weakness; unlike dermatomyositis, it is not usually associated with malignancy. However, there are a handful of case reports documenting polymyositis in patients with lymphoma, breast, lung, and bladder cancer. Here we report a case of metastatic pancreatic adenocarcinoma disguised by presenting as polymyositis. Clinical presentation, laboratory values, muscle biopsy, and imaging were all diagnostic of paraneoplastic polymyositis. The patient has significantly improved in symptoms are receiving systemic steroids and pancreaticoduodenectomy.

## Introduction

Polymyositis is an inflammatory myopathy that usually presents with bilateral proximal muscle weakness and inflammatory necrotizing infiltrates within striated muscle on histopathology [1]. There are three proposed classifications for polymyositis: primary idiopathic, childhood, and malignancy-associated [2]. Polymyositis is more often associated with Raynaud's phenomenon, interstitial lung disease, lupus, rheumatoid arthritis, scleroderma, and other connective tissue diseases [2]. However, inflammatory myopathies and their association with malignancy have been reported in the literature. The close temporal relationship between polymyositis onset and diagnosis of malignancy and improvement of symptoms with paraneoplastic syndrome treatment suggests a further association [3]. More specifically, polymyositis has been reported to occur as paraneoplastic syndromes in patients with lymphoma, breast, lung, and bladder cancers [4]. Paraneoplastic polymyositis is rarely associated with pancreatic adenocarcinoma, with less than a handful of documented cases. We present an unusual case of metastatic pancreatic cancer disguised within the paraneoplastic syndrome of polymyositis. 

Our case was presented as a poster at the Society of General Internal Medicine (SGIM) 2021 Annual Meeting.

## Case presentation

**Figure 1:**
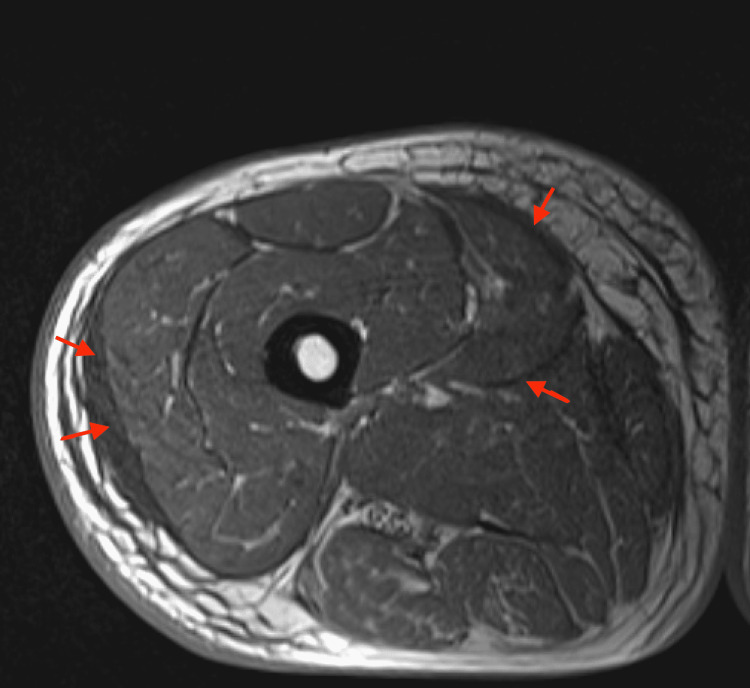
MRI of thigh T1 sequence, red arrows demonstrating diffuse subcutaneous and myofascial edema without focal abscess or necrosis.

**Figure 2:**
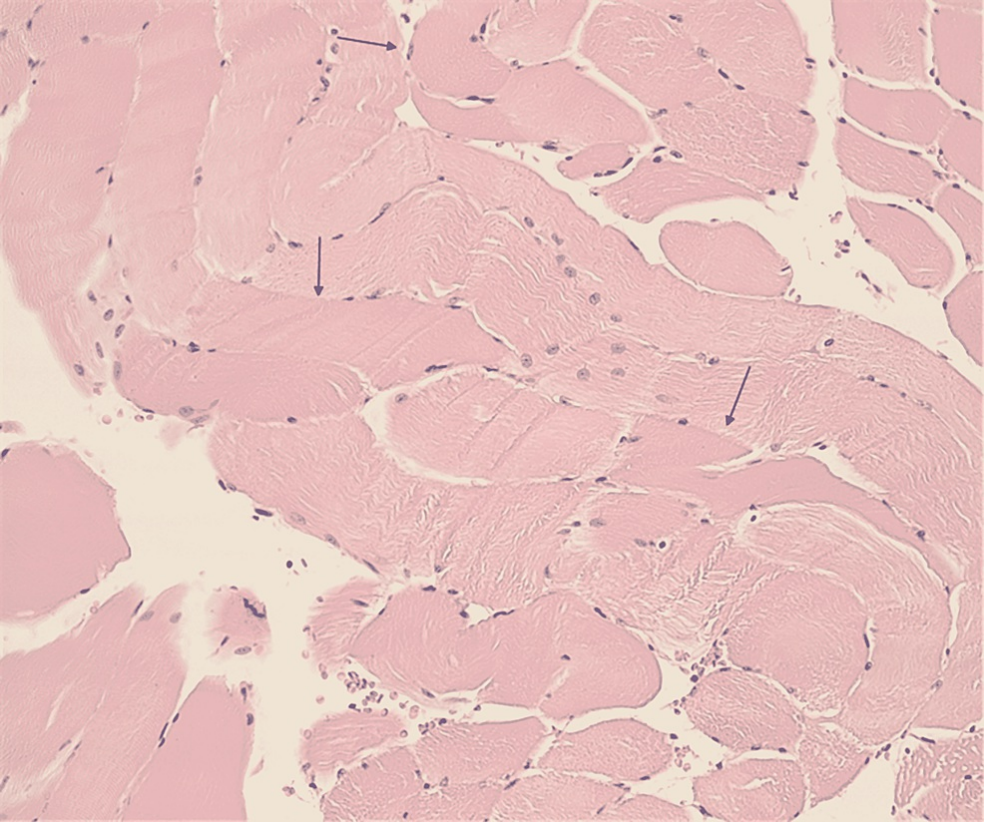
Biopsy from quadriceps muscle showing moderate variability in muscle size, scattered nuclear clumps, rare myofiber necrosis without inflammation; arrows point to degenerating skeletal muscle fibers (hypereosinophilic fibers)

An 81-year-old male with a history of hyperlipidemia on atorvastatin and no recent trauma presented with a two-week history of bilateral lower extremity myalgias and weakness. On presentation to the emergency room, the patient was alert, oriented, and vitally stable. Laboratory work-up was significant for an elevated creatinine kinase (CK) of 6059 U/l (normal value range 30-135 U/l), erythrocyte sedimentation rate (ESR) of 108 mm/h (normal value range 10-15 mm/h), and elevated C-reactive protein of 15.3 (normal value range <0.9 mg/dl). The patient was admitted to the hospital for treatment of acute rhabdomyolysis. The statin was discontinued and the patient was treated with intravenous crystalloids and solumedrol. Magnetic resonance imaging (MRI) of the right thigh revealed findings consistent with severe inflammatory myopathy with significant diffuse subcutaneous myofascial edema without focal abscess or necrosis (Figure 1). Muscle biopsy showed scattered nuclear clumps and rare myofiber necrosis without inflammation (Figure 2). Extensive antibody panel including autoantibodies against JO-1, PL-7, PL-12, EJ, OJ, SRP, MI-2, transcription intermediary factor 1-gamma (TIF1-γ) (P155/140), melanoma differentiation-associated protein 5 (MDA-5) (P140) (clinically amyopathic dermatomyositis (CADM)-140), nuclear matrix protein-2 (NXP-2) (P140), anti polymyositis/scleroderma (PM/SCL)-100 Ab, fibrillarin (U3 RNP), U2 snRNP Ab, Anti-U1-RNP Ab, KU, anti-Sjögren's-syndrome-related antigen A (Anti-SS-A) were all negative.

**Figure 3:**
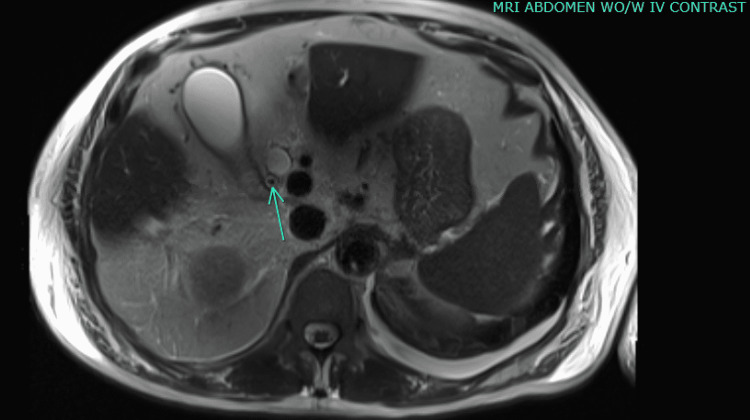
MRI abdomen with and without contrast with magnetic resonance cholangiopancreatography (MRCP) showing a 15 mm dilated distal common bile duct, 10 mm dilated pancreatic duct, and ampulla with a nonspecific 0.6 cm soft tissue density.

The patient's hospital course was complicated by sepsis secondary to cholangitis. Computed tomography (CT) of the abdomen was unremarkable; however, magnetic resonance cholangiopancreatography (MRCP) showed a 15 mm dilated distal common bile duct, 10 mm dilated pancreatic duct, and ampulla with a nonspecific 0.6 cm soft tissue density (Figure 3). CA 19-9 was elevated to 9298 U/ml. The patient underwent endoscopic retrograde cholangiopancreatography (ERCP) with sphincterotomy, which showed common bile duct (CBD) restriction, and diagnostic laparoscopy, which revealed liver lesions. CBD brushing and biopsy from liver lesions revealed the diagnosis of metastatic pancreatic adenocarcinoma.

The polymyositis was ruled to be a paraneoplastic presentation secondary to pancreatic adenocarcinoma and the diagnosis was confirmed with a muscle biopsy and highly compatible imaging findings. The patient was initially treated with systemic steroids (IV Solu-Medrol 20 mg daily for eight days), which had to be stopped as the hospital course was complicated with cholangitis. Ultimately, the patient underwent pancreaticoduodenectomy followed by palliative chemotherapy consisting of folinic acid, fluorouracil, and oxaliplatin (FOLFOX) for 12 cycles. He was able to move without muscle pain and CK elevation had been resolved in follow-up. Unfortunately, the patient developed neuropathy secondary to oxaliplatin and the chemotherapy regimen switched to 5-fluorouracil with leucovorin, then a short course of folinic acid, fluorouracil, and irinotecan (FOLFIRI). Due to tumour progression on prior treatments, the patient is currently on third-line palliative chemotherapy of gemcitabine and Abraxane.

## Discussion

In 1975, Bohan and Peter first established five classifications for inflammatory myopathies [5,6]. In 1991, Dalakas et al. [7] created the inflammatory idiopathy myopathy criteria, which established six classifications and included histopathological characteristics [5]. Unfortunately, features of myopathies are not specific and often overlap, exclusion data is vague, and a variety of extra-muscular manifestations can occur; thus, numerous individuals have established new criteria and classifications over the years [5]. The earliest reports of inflammatory myopathy associated with malignancy were in 1916 [1]. Polymyositis in the elderly population can present as a paraneoplastic syndrome and, if found, is strongly associated with underlying cancer. Dermatomyositis, on the other hand, is the subtype that has a stronger association with underlying malignancy but has been infrequently linked to pancreatic adenocarcinomas [8,9]. To date, only five detailed cases have been published in the literature describing paraneoplastic myopathies with pancreatic cancers (Table 1). Hill et al. completed an analysis of cancer sites for dermatomyositis and polymyositis among patients in Sweden, Denmark, and Finland [10]. Of the 115 dermatomyositis cases, five were associated with pancreatic cancer, and of the 95 polymyositis cases, one was associated with pancreatic cancer [10]. 

Syrios et al. and Amroun et al. reported similar cases of pancreatic adenocarcinoma-associated polymyositis in 2011 and 2012, respectively, where the inflammatory myopathy in their patients presented following the malignancy diagnosis, gemcitabine chemotherapy, and surgical resection [1,9]. It should be noted that gemcitabine-related myopathy cannot conclusively be ruled out. Our case, however, is unique as myositis was reported as the initial presenting symptom; thus, confounding elements such as chemotherapy administration do not exist.

Once there is clinical suspicion of polymyositis, patients should undergo a diagnostic workup. As concluded from the cases presented in Table 1, there is no consensus about specific testing. However, serum muscle enzymes, electromyography (EMG), and muscle biopsies are usually completed. Creatinine kinase may be increased up to 50x during active disease and is the most sensitive muscle enzyme marker [2,7]. Other makers that are increased are aspartate and alanine aminotransferases, lactate dehydrogenase, and aldolase [2,7]. Imaging can vary from ultrasound, CT, and MRI [2]. EMG will show fibrillations and repetitive complex discharges [7]. A muscle biopsy is necessary to establish this diagnosis, as only 30% of patients have positive antibody panels [1]. On histopathology, CD8/MHC-1 complexes, lymphocytic infiltrates surrounding and invading healthy muscle fibres, are indicative of polymyositis [7,9]. Malignancy can develop before or after myositis is diagnosed. The specific pathophysiology of malignancy-associated myositis is not well understood. The most widely held belief is that myositis is a paraneoplastic phenomenon triggered by autoimmunity. Compromised immune function, autoimmunity, or common environmental variables are also thought to play a role in the development of cancer-related myositis [11].

**Table 1:** Descriptive cases within the published literature of pancreatic malignancy disguised initially as myositis. M: male; F: female; CT: computed tomography; MRI: magnetic resonance imaging

Reference	Sex/Age	Symptoms	Biopsy/EMG	Imaging	Treatment
Padniewski et al. [4] 2021	M/66	generalized fatigue, progressive weakness, loose stools, weight loss	No/No	CT, MRI	steroids, chemotherapy
Amroun et al. [9] 2012	M/47	abdominal pain, jaundice, weight loss, asthenia	Yes/No	CT, MRI	chemotherapy, surgery
Siddiqui et al. [12] 2011	F/86	progressive proximal muscle weakness	Yes/No	CT	steroids, surgery
Syrios et al. [1] 2011	M/52	weight loss, recurrent pancreatitis	Yes/Yes	CT	steroids, chemotherapy, surgery
Kida et al. [13] 2007	M/53	not reported	Not reported	CT	steroids, chemotherapy, radiotherapy

In regard to the treatment of cancer-associated myositis, corticosteroids are the mainstay of therapy which is similar to idiopathic immune-mediated myositis treatment. Some authors suggest a three to four week taper of prednisone as first-line therapy [7]. Azathioprine and methotrexate may be started in conjunction with steroids if steroids become ineffective or if the patient has worsening symptoms [2,7]. Intravenous immunoglobulin, cyclosporine, mycophenolate mofetil, and cyclophosphamide are usually considered as later options [7]. It is important to consider underlying comorbidities when determining treatment. For example, our patient completed systemic steroids and had a pancreaticoduodenectomy followed by palliative chemotherapy. All of the patients reported in Table 1 received steroids except for one. It has been reported that lack of response to steroid therapy may indicate underlying malignancy, thus, indicating the need for further investigation [4]. Furthermore, treatment of underlying malignancy may improve myopathy symptoms.

## Conclusions

Polymyositis has been referred to as a diagnosis of exclusion or mimicker, which can lead to delayed or misdiagnosis. Our case demonstrates the necessity of maintaining clinical suspicion of underlying malignancies in patients with inflammatory myopathies. This awareness can play a role in early detection and diagnosis and thereby impact health outcomes of this lethal disease.
